# Spirituality, dimensional autism, and schizotypal traits: The search for meaning

**DOI:** 10.1371/journal.pone.0213456

**Published:** 2019-03-08

**Authors:** Bernard Crespi, Natalie Dinsdale, Silven Read, Peter Hurd

**Affiliations:** 1 Department of Biological Sciences, Simon Fraser University, Burnaby, British Columbia, Canada; 2 Department of Psychology, University of Saskatchewan, Saskatoon, Saskatchewan, Canada; 3 Department of Psychology and Centre for Neuroscience, University of Alberta, Edmonton, Alberta, Canada; University of La Rioja, SPAIN

## Abstract

The relationships of spirituality with human social cognition, as exemplified in autism spectrum and schizophrenia spectrum cognitive variation, remain largely unstudied. We quantified non-clinical levels of autism spectrum and schizotypal spectrum traits (using the Autism Quotient and the Schizotypal Personality Questionnaire-Brief Revised) and dimensions of spirituality (using the Hardt Spirituality Questionnaire) in a large sample of undergraduate students. We tested in particular the hypothesis, based on the diametrical model of autism and psychosis, that autism should be negatively associated, and positive schizotypal traits should be positively associated, with spirituality. Our primary findings were threefold. First, in support of the diametric model, total Spirituality score was significantly negatively correlated with total Autism Quotient score, and significantly positively correlated with Positive Schizotypal traits (the Schizotypal Personality Cognitive-Perceptual subscale), as predicted. Second, these associations were driven mainly by opposite patterns regarding the Search for Meaning Spirituality subscale, which was the only subscale that was significantly negatively associated with autism, and significantly positively associated with Positive Schizotypal traits. Third, Belief in God was positively correlated with Positive Schizotypal traits, but was uncorrelated with autism traits. The opposite findings for Search for Meaning can be interpreted in the contexts of well-supported cognitive models for understanding autism in terms of weak central coherence, and understanding Positive Schizotypal traits in terms of enhanced salience.

## Introduction

The psychological bases and correlates of religious beliefs, participation and experience have recently been subject to an increasing depth and breadth of empirical work [1]. Most of these studies have focused on how variation in aspects of human cognition, sociality, and personality is related to variation in aspects of religiosity, among non-clinical populations. These analyses have revealed important insights into the psychological underpinnings of religiosity, including: (1) negative associations of religiosity and belief in God with physical-world beliefs, interests, and knowledge [2,3] and analytical thinking more generally [2]; and (2) positive associations of religiosity with empathizing, as well as with social, moral and emotional cognition [4]; [5].

Given important roles for social cognition in religious thinking and behavior, a closely related stream of research has addressed the presence, nature and patterns of religiosity among individuals characterized by clinical or non-clinical psychiatric conditions that involve diversity and extremes of sociality (e. g., [2,4,6]). Two conditions in particular, the autism spectrum and the schizophrenia or schizotypy spectrum, engender divergence from non-clinical individuals in aspects of social cognition, including imagination, mentalizing and theory of mind, inference of agency, and magical thinking, that are expected to impact upon religious beliefs and thinking. Aspects of religious cognition in these conditions may thus be informative concerning both the psychological architectures of autism and schizotypy and cognition in non-clinical individuals. In particular, the autism spectrum has been considered to centrally involve less-mentalistic and more-mechanistic cognition than in non-clinical individuals, whereas schizotypal traits (especially positive schizotypal traits, which involve the emergence of novel psychosis-related cognitive and perceptual phenomena) engenders the opposite, cognition that is more mentalistic and less mechanistic [7,8]. As such, these two conditions are expected, from relevant theory and previous empirical work, to exhibit opposite relationships with aspects and correlates of religiosity, with autism showing inverse associations and schizotypal traits showing associations that are positive.

Schizotypy represents a latent multidimensional construct associated with putative liability to schizophrenia [9,10]; it exists along a continuum of expression from low to high, and can be quantified by assessing levels of schizotypal traits [11–13]. Levels of schizotypal traits, especially positive schizotypal traits that include magical thinking, unusual perceptions, and ideas of reference (exaggerated belief that innocuous events refer significantly to oneself, especially in the context of paranoia), have indeed been demonstrated to be positively associated with measures and aspects of religiosity and paranormal thinking, including for example belief in God and powerful supernatural beings [6,14–19]. These associations have been attributed to a variety of psychological factors linked with positive schizotypal traits, including a highly-developed sense of agency, over-developed mentalistic thought and mental state projection, a less-analytical cognitive style, unusual perceptions grading into hallucinations, and ideas and delusions of reference, whereby external events are over-interpreted and misinterpreted in terms of direct personal significance (e. g., [20]). Despite the clear and strong links of positive schizotypal traits and schizophrenia with manifestations of religiosity and the supernatural, schizotypal traits and schizophrenia are not positively associated with participation in traditional religious institutions [21,22], probably due at least in part to the reduced social, interpersonal motivation and abilities also found in this set of conditions and, for schizotypal traits, the lack of centrality for personal religious experiences in traditional religions.

Empirical research on the autism spectrum in relation to religiosity has focused mainly on the associations of autism diagnosis, and autism traits in non-clinical populations, with belief in God, and on associations of mentalizing ability with religiosity. Norenzayan et al. [4] presented evidence that individuals with autism expressed less belief in a 'personal God' than typical controls, a difference that was mediated by reduced mentalizing in the autism group. By contrast, Reddish, Tok, and Kundt [23] reported differences between individuals with autism and controls with regard to mentalizing abilities (using false-belief tasks and Happé's Strange Stories test), but virtually no differences on a set of measures of religiosity, including belief in God; moreover, associations of mentalizing with aspects of religiosity were generally non-significant, weak, and negative. Jack et al. [5] likewise found no evidence of associations between mentalizing and religious belief, using nine mentalizing measures. They reported instead that religious belief was selectively associated with high moral and emotional concern, and with low levels of analytical thinking. High levels of analytical thinking in autism may thus be expected to mediate, in part, any reduction in religiosity found among these individuals, given that an analytical cognitive style is negatively associated with religious and paranormal beliefs [24]; see also [3,25]. More broadly, the autism spectrum involves a considerable suite of traits that each might be expected to engender reduced, or different, belief in God or religion, including: (1) reduced social connectedness and theory of mind;, (2) restricted non-social, mechanistic, and analytical interests; (3) reduced empathizing and high systemizing; (4) reduced perception and inference of agency; (5) increased focus on details and parts rather than the 'big picture'; and (6) reduced moralizing, often expressed in utilitarian judgements (e. g., [26,27]). However, the relative roles of these factors remain to be discerned.

Two studies to date have analyzed autistic and schizotypal traits together, in relation to aspects of religiosity. Thus, Gray, Jenkins, Heberlein, and Wegner [28] found that scoring highly on the Autism Quotient (AQ) was associated with reduced perception of agency in humans, and that scoring highly on the Schizotypal Personality Questionnaire (SPQ) Cognitive-Perceptual subscale (positive schizotypal traits) was linked to 'seeing mind where there is none' by ascribing mind to trees, the dead, and God, and to assignment of more agency than normal to robots and dogs, which can jointly be interpreted as belief that all entities exhibit forms of consciousness. These findings highlight central cognitive differences in mind and agency perceptions between autism and positive schizotypal traits, especially with regard to mentalistic thought.

Lindeman and Lipsanen [2], also using the AQ and a version of the SPQ, found that religiosity was significantly positively associated with schizotypal traits, but significantly negatively associated with autistic traits, although the proportion of variation accounted for was low. In their analysis, the most consistent difference between religious believers and non-believers was 'interests and skills needed to understand the physical world', a finding consistent with a notable body of previous work, as well as with theory of these conditions as cognitively opposite to one another [5,7].

Taken together, the bulk of the evidence to date therefore suggests reduced religiosity on the autism spectrum, and increased religiosity on the schizophrenia spectrum, especially with regard to positive schizotypal traits and positive schizophrenia symptoms. These findings represent important first steps towards understanding the psychological bases of religious belief and experience broadly construed, but they exhibit some notable limitations. Of these, one of the most important is that almost all of the work has focused on aspects of religion considered from modern, Western perspectives. Recent large-scale religions, and modern beliefs in and conceptions of God, are sufficiently recent phenomena that they represent evolutionary novelties, to which humans are not predicted to be adapted in any specific genetic, neurological or cognitive sense (e. g., [26]). Indeed, current conceptions of the world as divisible into 'natural' and 'supernatural' beings and events are a recent phenomenon: hunter-gatherer and small-scale societies do not make this distinction, and live in a natural world imbued with spirituality. How might spirituality, then, be related to psychological predicates?

Lindeman, Svedholm-Häkkinen, and Lipsanen [29] found, in a large sample of typical Finnish individuals, that religious beliefs, paranormal beliefs, and belief in supernatural purpose were highly predicted not by mentalizing abilities, but by what they call 'core ontological confusions', considered as cognitive biases that involve ascribing mental states and agency to entities in the physical world. To a close approximation, such 'core ontological confusions' represent forms of spiritual animism, the 'animation' with spirits of all salient phenomena in the natural world, be they rocks, water holes, or goanna lizards. These authors also found similarly strong prediction of religious beliefs, paranormal beliefs, and belief in supernatural purpose from 'hyper-empathizing' (high empathizing combined with low systemizing), a construct also linked positively with aspects of subclinical psychosis traits by Brosnan, Ashwin, Walker, and Donaghue [30]. Most directly, Willard and Norenzayan [31] analyzed the psychological correlates of being 'spiritual but not religious', finding that such individuals scored more highly for positive schizotypy than did religious individuals and controls, as well as endorsing a higher incidence of paranormal beliefs and supernatural, mystical experiences.

These findings suggest that spirituality represents a form of human cognitive-affective experience that is both centrally important and substantially distinct from religiosity, and from the supernatural, as well as representing an ancestral, long-term, and ongoing (in many societies) state of cognition and experience. Under a spiritual world-view, then, natural and supernatural phenomena and events are not separated, one engages in searches for meaning within one's life, one's group, one's culture, and the universe most broadly, and one focuses on personal beliefs and experiences concerning existence, states of being, and one's relationship with the world and its constituents. A god or gods may be considered to exist, but not in the context of 'institutionalized' religions beyond the belief systems of one's own tribe, for small-scale societies. Spirits, 'animism', souls of ancestors and others, and origin stories, while they can certainly be viewed as 'cognitive biases' or 'ontological confusions' by scientific world-views, can also represent, under spiritual world-views, important existential features of cognitive as well as external landscapes.

Most previous studies of the psychology of religiosity in non-clinical individuals, or individuals in relation to the autism spectrum or schizotypy spectrum, have thus centered on facets of contemporary religion, which may include for example belief in a single god. In this context, and to operationalize the distinctions applied here, religiosity can be considered to comprise two primary, inter-connected components, (1) supernatural phenomena, including ideas concerning creation of the world and (2) moral precepts. These religious concepts are now seen most prominently in Christianity, Islam, and Judaism. However, the current focus on contemporary religious groups and institutions presents challenges for interpretation of the social, cultural, neurological and genetic contexts under which cognition related to religiosity evolved, given that the primary environments for the evolution of the biological and cultural underpinnings of religiosity and related phenomena must have been pre-historical, and comparable to those found in hunter-gatherer and other small-scale societies.

In human hunter-gatherer and other small-scale groups, and as inferred from phylogenetic studies of diverse human cultures [32], the ancestral human condition for 'religiosity' thus reflects spirituality, which, from cultural and empirical perspectives, is profoundly different from contemporary religion. As noted above, under traditional spiritualistic cognition, the natural and supernatural worlds are in no way distinct, and places, objects, and living creatures including humans and selves are animated by spirits or souls, that imbue them with living properties and meanings. The primary upshot of these considerations is that the human mind evolved predominantly in the context of what we now call spirituality, rather than what we call religion [32]. As such, analyzing the psychological underpinnings of human spirituality, in relation to the cognitive variation expressed in autistic and schizotypal thinking, may provide novel and useful insights into spirituality and religion.

What, then, are the psychological correlates and underpinnings of human spirituality, and how do they relate to the autism spectrum and schizotypal spectrum? In this article we have collected self-report data, from a large, healthy, non-clinical population, on (1) a measure of spiritual cognition, and its major components [33]; and (2) positions on metrics of autism spectrum (the Autism Quotient AQ: [34]) and schizotypal spectrum (the Schizotypal Personality Quotient Brief-Revised SPQ-BR: [35]) cognition. The main goal of this study is to determine how autism spectrum and positive schizotypal spectrum traits are related to spirituality and its dimensions. Our overarching prediction is that spirituality, in whole or for key components, should be positively associated with positive schizotypal traits, and negatively associated with autism spectrum traits.

## Methods

### Participants and ethics approval

Data was collected from 1194 undergraduate students (731 females, and 463 males, mean ages 19.4 for both; ethnicities Caucasian 42%, Asian 35%, Others 23%) at the University of Alberta. Biological sex, rather than (culturally-defined) gender was reported, for comparison with previous relevant studies, in the context of statistical differences and adjustments. Given the non-clinical nature of the population, subjects were not questioned concerning psychiatric diagnoses. The work was approved by Human Research Ethics at University of Alberta and by the Simon Fraser University Research Ethics Board, and all subjects gave written informed consent before participating in the study.

### Psychological measures

The Hardt Spirituality Questionnaire (HSQ) [33] was used to assess self-report spirituality. The HSQ is a 20-item questionnaire with responses on a 5-point Likert scale of how true the question was perceived to be in relation to the respondent. Four subscales are represented: (1) Belief in God (any God, and having a relationship with them involving trust, love, faith and friendship); (2) Search for Meaning (centered on the quest for existential meaningfulness, searching for spiritual insights, an open mind and an expanded soul); (3) Mindfulness (conscious meta-cognitive perception of others and one's environment, empathy, and attentive self-awareness); and (4) Security (safety and trust in the world, and feeling at home). Total Spirituality was the sum of the subscales. Cronbach’s alpha values (calculated using the alpha() function from the R ‘psych’ library) for these subscales were 0.94, 0.71, 0.72 and 0.73 respectively, and the overall alpha value was 0.88.

The Autism Spectrum Quotient (AQ) [34] was used to quantify endorsement of autism spectrum traits. The AQ is a 50-item self-report questionnaire that assesses autism-related traits across five domains: (1) Social Skills; (2) Communication; (3) Attention to Detail; (4) Attention Switching; and (5) Imagination. Higher autism traits are thus quantified as lower social skills and communication, higher attention to detail, less attention switching, and reduced imagination (mainly social imagination). Responses range from ‘definitely agree’ to ‘definitely disagree’ on a 4-point Likert scale; Total Autism score is the sum of the subscales. Cronbach’s alpha values (calculated using the alpha() function from the R ‘psych’ library) for these subscales were 0.69, 0.52, 0.55, 0.48, and 0.44 respectively.

Schizotypal traits were quantified using the Schizotypal Personality Questionnaire-Brief Revised (SPQ-BR) [35]. This questionnaire includes 32 items in a 5-point Likert-scale format, that includes seven subscales: (1) Constricted Affect; (2) Social Anxiety; (3) Magical Thinking; (4) Unusual Perceptions; (5) Ideas of Reference; and (6) Eccentric Behavior; and (7) Odd Speech. Total Schizotypy is the total of all seven subscales. Cronbach’s alpha values (calculated using the alpha() function from the R ‘psych’ library) for these seven subscales were 0.81, 0.84, 0.76, 0.52, 0.72, 0.85 and 0.57 respectively. There are also three higher-level subscales: 'Interpersonal' (made up of 1+2), 'Cognitive-Perceptual' (3+4+5), which represents Positive Schizotypal Traits, and 'Disorganized' (6+7). We focus here on Positive Schizotypal Traits, based on both theoretical considerations and previous relevant empirical work showing that only this dimension of schizotypy is related to aspects of religiosity. Individuals with missing data, or scores that were outliers for the required range, for any of the questionnaires were excluded from analysis.

### Analyses

Analyses were conducted in R (v3.4.3). Sex effects in Table 1 were tested with Welch’s t-tests, and effect sizes were measured using Cohen’s d with 95% confidence intervals calculated using 1000 bootstrap resamplings [36].

**Table 1:** Sex differences in the phenotypes analyzed.

	Male Mean (SD)		Female Mean (SD)		t	df	p	p	d'
								(with FDR adj.)	Effect size)
**Spirituality Total**	3.35	(0.64)	3.50	(0.64)	**-4.01**	957.4	<0.0001	<0.0005	-0.239
Belief in God	2.70	(1.35)	2.98	(1.35)	**-3.53**	984.9	<0.0005	<0.002	-0.210
Search for Meaning	3.37	(0.74)	3.47	(0.74)	-2.18	960.6	0.03	0.06	-0.130
Mindfulness	4.14	(0.53)	4.29	(0.53)	**-5.05**	893.7	<0.0001	<0.0001	-0.304
Security	3.19	(0.76)	3.26	(0.76)	-1.58	939.4	0.1	0.2	-0.094
**Autism Total**	18.51	(5.78)	18.44	(5.78)	0.19	963.9	0.9	0.9	0.011
Social Skills	2.51	(2.24)	2.65	(2.24)	-1.11	965.3	0.3	0.3	-0.066
Attention Switching	5.01	(1.94)	5.17	(1.94)	-1.40	989.9	0.2	0.2	0.083
Attention to Detail	5.52	(2.07)	5.67	(2.07)	-1.24	993.7	0.2	0.3	-0.073
Communication	2.65	(1.88)	2.62	(1.88)	0.27	981.4	0.8	0.8	0.016
Imagination	2.83	(1.85)	2.34	(1.85)	**4.75**	887.9	<0.0001	<0.0001	0.286
**Positive Schizotypal (Cognitive-Perceptual)**	36.26	(7.45)	37.70	(7.45)	**-3.15**	1041.0	<0.002	<0.005	-0.186
Ideas of Reference	17.57	(4.20)	18.11	(4.20)	-2.19	971.9	0.03	0.06	-0.130
Magical Thinking	7.87	(3.36)	9.11	(3.36)	**-5.94**	1059.5	<0.0001	<0.0001	-0.348
Unusual Perceptions	10.82	(2.64)	10.47	(2.64)	2.10	1046.4	0.0356	0.0594	0.124

Results significant after FDR adjustment are in boldface.

Independent analyses of covariance between spirituality (and its subscales) and autism and positive schizotypy (and their subscales) were conducted including sex as a cofactor using type-II sums of squares to test for significant interactions of sex with the other variables using the Anova() function from the “car” library [37]. In only two of these 55 analyses did sex show a significant interaction effect, demonstrating that there were generally no sex differences in the relationships of sex with other factors. We therefore present the main effects for sex in these analyses. The two significant cases will be discussed specifically, to the limited extent that the effects are relevant.

Partial correlation coefficients, in multiple regressions of spirituality measures on sex, autism and positive schizotypy scales, were calculated using the pcor() function in the “ppcor” library [38].

## Results

### Sex differences

We found no sex differences in Total Autism scores, while Positive Schizotypal traits and Total Spirituality all showed significant female biases (Table 1), as found in previous studies (e. g., [39–42]). The three subscales of Positive Schizotypal traits showed differing sex biases, with women scoring significantly higher on Ideas of Reference and Magical Ideation, and significantly lower than men on Unusual Perceptions. Only one of the four subscales of autism, Imagination, showed a strong male bias. The sex difference in spirituality was evident in three of the four subscales, with women scoring higher for Belief in God, Search for Meaning and Mindfulness, but not Security (Table 1). Most of these subscale sex differences have small effect sizes, but two subscales of religiosity, Belief in God and Mindfulness, and one subscale each from the autism and Positive Schizotypal traits assays, Imagination and Magical Ideation respectively, are considerably stronger (d’ stronger than 0.2, Table 1). Sex was therefore included as a covariate as we examined the relationships between spirituality and these spectrum traits.

### Bivariate associations

We examined the bivariate associations between Total Spirituality scores, and the predictor variables of Total Autism, and Positive Schizotypal traits including sex as a covariate (Table 2). Total Autism was negatively associated with Total Spirituality, while Positive Schizotypal traits showed a positive association (Table 2). The p = 0.09 sex interaction effect between Positive Schizotypal traits and Total Spirituality was not due to opposing effects in the two sexes, but to the relatively stronger relationship in males. The positive correlation between these two traits was thus significant in both sexes, but relatively stronger in males. In contrast to the positive relationship of Total Spirituality with Positive Schizotypal traits, Total, Interpersonal, Disorganized Schizotypal traits were each negatively related to Total Spirituality (r = -0.07, p = 0.024; r = -0.23, p <0.00000001; r = -0.08, p = 0.0052 respectively). These results support the hypothesis that spirituality is positively related exclusively to the positive dimension of schizotypal traits, as noted above in previous studies.

**Table 2:** Correlations of total spirituality with autism and schizotypal traits, and results from ANCOVAs testing for sex by trait interaction effects.

	r	p	p	p
	Total Spirituality	Total Spirituality	FDR Adj.	Total Spirituality by Sex
**Autism Total**	**-0.16**	0.00002	<0.00001	0.879
Social Skills	**-0.23**	<0.00001	<0.00001	0.353
Attention Switching	-0.03	0.6	0.6	0.772
Attention to Detail	0.04	0.5	0.6	0.456
Communication	**-0.16**	<0.00001	0.00002	0.529
Imagination	**-0.09**	0.03	0.049	0.718
**Positive Schizotypy (Cognitive-Perceptual subscale)**	**0.14**	0.005	0.02	0.0911
Ideas of Reference	-0.03	0.3	0.4	0.543
Magical Thinking	**0.22**	<0.00001	<0.00001	0.0313
Unusual Perceptions	**0.13**	0.005	0.02	0.131

Results significant after FDR adjustment are in boldface.

We further analyzed the bivariate associations between the subscales of Total Spirituality and each of the subscales of the spectrum trait (Tables 3–6). In only two of these 55 analyses did sex show a significant interaction effect: (1) between Magical Thinking and Total Spirituality, where significant positive correlations between these traits in were stronger in males than females, leading to a significant interaction term for sex, and (2) between Magical Thinking and Mindfulness, where males showed a significant positive effect and females showed no effect. These results demonstrate that while there may be sex differences in these psychological traits, there were generally no sex differences in the relationships between them.

**Table 3:** Correlations of belief in god with autism and schizotypal traits, and results from ANCOVAs testing for sex by trait interaction effects.

	r	p	p	p
	Belief in God	Belief in God	FDR Adj.	Belief in God by Sex
**Autism Total**	-0.02	0.3	0.4	0.398
Social Skills	**-0.10**	0.008	0.02	0.981
Attention Switching	0.06	0.5	0.6	0.115
Attention to Detail	0.01	0.7	0.8	0.986
Communication	-0.03	0.1	0.2	0.354
Imagination	0.01	0.8	0.8	0.982
**Positive Schizotypy (Cognitive-Perceptual subscale)**	**0.15**	0.0009	0.003	0.101
Ideas of Reference	0.06	0.3	0.4	0.383
Magical Thinking	**0.18**	0.00002	0.00006	0.129
Unusual Perceptions	**0.11**	0.02	0.04	0.201

Results significant after FDR adjustment are in boldface.

**Table 4:** Correlations of search for meaning with autism and schizotypal traits, and results from ANCOVAs testing for sex by trait interaction effects.

	r	p	p	p
	Search for Meaning	Search for Meaning	FDR Adj.	Search for Meaning by Sex
**Autism Total**	**-0.10**	0.03	0.04	0.420
Social Skills	**-0.14**	0.002	0.005	0.292
Attention Switching	0.01	0.8	0.8	0.322
Attention to Detail	0.07	0.2	0.3	0.285
Communication	**-0.10**	0.007	0.01	0.887
Imagination	**-0.12**	0.02	0.04	0.231
**Positive Schizotypy (Cognitive-Perceptual subscale)**	**0.25**	<0.00001	<0.00001	0.413
Ideas of Reference	0.07	0.07	0.1	0.955
Magical Thinking	**0.28**	<0.00001	<0.00001	0.201
Unusual Perceptions	**0.23**	<0.00001	<0.00001	0.408

Results significant after FDR adjustment are in boldface.

**Table 5:** Correlations of mindfulness with autism and schizotypal traits, and results from ANCOVAs testing for sex by trait interaction effects.

	r	p	p	p
	Mindfulness	Mindfulness	FDR Adj.	Mindfulness by Sex
**Autism Total**	**-0.24**	<0.00001	<0.00001	0.566
Social Skills	**-0.25**	<0.00001	<0.00001	0.705
Attention Switching	-0.11	<0.05	0.07	0.0701
Attention to Detail	0.08	0.05	0.08	0.649
Communication	**-0.24**	<0.00001	<0.00001	0.554
Imagination	**-0.18**	<0.00001	0.00003	0.896
**Positive Schizotypy (Cognitive-Perceptual subscale)**	-0.04	0.04	0.07	0.0871
Ideas of Reference	**-0.09**	0.006	0.01	0.614
Magical Thinking	-0.02	0.5	0.5	0.0142
Unusual Perceptions	0.00	0.4	0.5	0.173

Results significant after FDR adjustment are in boldface.

**Table 6:** Correlations of security with autism and schizotypal traits, and results from ANCOVAs testing for sex by trait interaction effects.

	r	p	p	p
	Security	Security	FDR Adj.	Security by Sex
**Autism Total**	**-0.25**	<0.00001	<0.00001	0.355
Social Skills	**-0.28**	<0.00001	<0.00001	0.0550
Attention Switching	-0.11	0.04	0.07	0.0879
Attention to Detail	0.00	0.5	0.5	0.227
Communication	**-0.22**	<0.00001	<0.00001	0.908
Imagination	**-0.11**	0.006	0.01	0.999
**Positive Schizotypy (Cognitive-Perceptual subscale)**	-0.04	0.1	0.2	0.431
Ideas of Reference	**-0.20**	<0.00001	<0.00001	0.849
Magical Thinking	**0.12**	0.01	0.03	0.119
Unusual Perceptions	0.03	0.8	0.8	0.295

Results significant after FDR adjustment are in boldface.

In the bivariate analyses of spirituality subscales, only Search for Meaning (Table 4) showed the same pattern of significant negative association with Total Autism, and significant positive association with Positive Schizotypal traits, as was found for Total Spirituality. Mindfulness (Table 5) and Security (Table 6) both showed strong, significant negative correlations with Total Autism but essentially no relationship with Positive Schizotypal traits; by contrast, Belief in God (Table 3) showed a significant positive correlation with Positive Schizotypal traits, but no association with Total Autism.

### Multivariate associations

To partition independent effects of sex, autism traits and Positive Schizotypy traits on aspects of spirituality, we conducted multiple regressions of Total Spirituality, and each of the four spirituality subscales, on sex and each AQ and SPQ lower-level subscale (Table 7). The coefficients were not substantially changed in relation to the bivariate results shown in Tables 2–6, with (1) Total Spirituality predicted mainly by sex, Social Skills, and Magical Thinking; (2) Belief in God predicted from sex, Social Skills and Magical Thinking; (3) Search for Meaning predicted from Social Skills, Communication, Imagination, Magical Thinking, and Unusual Perceptions (the same as in the bivariate analysis); (4) Mindfulness predicted from sex, Social Skills, Communication, and Imagination; and (5) Security predicted from Social Skills, Ideas of Reference, and Magical Thinking.

**Table 7:** Partial regression coefficients from multiple regressions of autism and schizotypy subscale traits on aspects of spirituality.

	Total Spirituality		Belief in God		Search for Meaning		Mindfulness		Security	
	Partial r	p	Partial r	p FDR Adj.	Partial r	p	Partial r	p	Partial r	p
		FDR Adj.				FDR Adj.		FDR Adj.		FDR Adj.
Sex	**-0.09**	0.004	**-0.08**	0.01	-0.03	0.5	**-0.14**	0.00001	-0.04	0.2
AQ-Social Skills	**-0.16**	<0.00001	**-0.10**	0.002	**-0.08**	0.02	**-0.13**	0.00007	**-0.19**	<0.00001
AQ-Attention Switching	**0.08**	0.02	**0.09**	0.005	0.05	0.2	0.01	0.7	0.03	0.4
AQ-Attention to Detail	-0.01	0.9	-0.02	0.5	0.01	0.8	0.06	0.05	-0.02	0.5
AQ-Commun-ication	**-0.07**	0.03	-0.03	0.5	**-0.08**	0.02	**-0.10**	0.002	-0.05<	0.2
AQ-Imagina-tion	0.03	0.4	0.04	0.3	**-0.07**	0.02	**-0.11**	0.0003	-0.03<	0.4
SPQ-Ideas of Reference	**-0.07**	0.04	0.00	0.9	-0.01	0.7	-0.04<	0.2	**-0.19**	<0.00001
SPQ-Magical Thinking	**0.19**	<0.00001	**0.15**	<0.00001	**0.22**	<0.00001	0.02	0.6	**0.15**	<0.00001
SPQ-Unusual Perceptions	**0.09**	0.004	0.05	0.2	**0.15**	<0.00001	0.02	0.5	0.06	0.06

Results significant after FDR adjustment are in boldface.

## Discussion

We have conducted the first analyses of both non-clinical autism spectrum traits and positive-schizotypal spectrum traits in relation to aspects of human spirituality. The main findings are threefold.

First, as predicted under the hypothesis that autism and positive schizotypy represent opposite psychological dimensions, Total Spirituality score was significantly negatively correlated with Total Autism Quotient score, and significantly positively correlated with Positive Schizotypal traits (the Schizotypal Personality Cognitive-Perceptual subscale). For autism, this overall negative association was mediated predominantly by effects from the Social Skills, Communication, and Imagination subscales, as might be expected if spirituality and religiosity involve, in part, imaginative social connectedness (e. g., [19,43]). By contrast, for Positive Schizotypal traits, the effects of variation in Magical Thinking predominated. This finding implies that magical thinking concerning supernatural entities and events may most-directly mediate links of Total Spirituality with Positive Schizotypal traits, again implicating imagination as an especially relevant psychological domain (e. g., [44]: pp. 4, 161). Spiritual, religious, supernatural, and paranormal experience have also been linked with higher scores on measures of positive schizotypy in previous studies (e. g., [6,15,18,45]); as also noted above, Willard and Norenzayan [31] showed in particular that individuals who were more spiritual (but not religious) scored higher on positive schizotypal traits, using an earlier version of the SPQ [46].

The signs and magnitudes of associations of the four spirituality components with subscales of autism and positive schizotypal traits appear to make general intuitive sense, in that (1) higher Belief in God is linked with better Social Skills and higher levels of Magical Thinking, as well as female sex, which fits with the well-established female bias in spirituality and religiosity (e. g., [40]); (2) higher Search for Meaning is associated with better Social Skills and Imagination, and higher levels of Magical Thinking and Unusual Perceptions; (3) higher Mindfulness is connected with better Social Skills, Communication, and Imagination (less-autistic traits for all three), and female sex; and (4) higher Security is linked with better Social Skills, Communication, and Imagination, lower paranoia (Ideas of Reference), and higher Magical Thinking (Tables 2 and 3). Overall, higher Social Skills combined with higher Magical Thinking appear to be most predictive of the spiritual phenotypes analyzed here (applying overall, and for 3 of 4 of the subscales), which is interesting in the context of spirituality involving social connectedness with what are, from modern Western views, 'supernatural' entities, objects, or states.

Second, the opposite associations of spirituality with Total Autism versus Positive Schizotypal traits were evident only in diametric patterns for the Search for Meaning spirituality subscale, which was significantly negatively associated with social and imaginative subscales of autism (and Total Autism), and significantly positively associated with Positive Schizotypal traits. Indeed, none of the three spirituality subscales other than Search for Meaning showed any trace of this diametric pattern.

The specificity of the diametric results to Search for Meaning is intriguing in the context of influential theories for explaining the psychological bases of autism and positive schizotypal traits (and their direct extension, psychosis in schizophrenia). Thus, for autism, Happé [47] and Happé & Frith [48] describe the concept of Weak Central Coherence as a predominant causal factor in autistic cognition; by this theory, individuals with autism exhibit a reduced 'drive for meaning' and a lesser tendency to 'see the big picture' in extracting global, high-level form and meaning from both the world and their thoughts. In principle, then, a less-developed drive for meaning in everyday activities and events may extend to the spiritual domain, where drive for meaning and central coherence would be required to seek, and find, personal spiritual significance in the world; by contrast, in contemporary large-scale religions, 'meaning' is directly provided. This hypothesis could be evaluated more directly by measuring performance on task-based correlates of central coherence, such the embedded figures test [47], in relation to spiritual searches for meaning.

For positive schizotypy and psychosis, a central framework for psychological and neurochemical understanding derives from the concept of salience (i. e., relevance, meaning or significance), whereby relatively high dopaminergic states lead to aberrant assignment of importance to particular experiences (leading to delusions) and internal representations (appearing as hallucinations) [20,49]. Indeed, the prominent schizophrenia researcher van Os [50] suggested that this condition be renamed as 'salience syndrome', given the centrality of dysregulated over-attribution of meanings in schizophrenia and psychosis more generally. In this context, increased spiritual search for meaning in high positive schizotypal individuals may represent a manifestation of upregulated salience, with non-clinical psychosis-related experiences (e. g., mild delusions and unusual perceptual experiences, magical thinking, and highly-developed imagination) as central forms of expression. Across studies, 20–70% of all delusions experienced by individuals with schizophrenia spectrum disorders have clear religion-related content, manifesting especially as forms of grandiosity and megalomania [16,51,52]. High schizotypal traits have also been associated with a tendency to perceive meaning where none exists, in experimental tests using animated shapes and responses to short stories [53]. A hypothesis based on increased salience in high schizotypy would benefit from psychological tests focused more specifically on aspects of salience in relation to spiritual search for meaning, as well as tests relating dopaminergic neurotransmission to this aspect of spiritual cognition and experience (e. g., [54]).

Third, we found that Belief in God was positively correlated with Positive Schizotypal traits, but was uncorrelated with autism traits. An association of Positive Schizotypal traits with Belief in God, in the context of spirituality (separate from religious tradition), was also reported by Willard and Norenzayan [31]. Some evidence supports a hypothesis of reduced belief in a personal God among individuals with autism or more autism-related traits, based on constraints imposed by a less-developed theory of mind [4]; by contrast, Jack et al. [5] describe evidence that belief in God is predicted by moral concerns and more-intuitive, less-analytical cognition, but not by theory of mind abilities per se. Persons with high-functioning autism have also been reported to exhibit lower levels of traditional religiosity, higher levels of atheism and agnosticism, and a greater tendency to construct their own belief-related systems [55]. The degree to which conceptions regarding spiritual or religious cognition, and God, differ between non-clinical individuals and individuals with autism or high levels of autism traits, remains to be determined. By some accounts (e. g., [56]), spirituality and religiosity in autism are expected to differ qualitatively from traditional norms, being more analytical, mechanistic, and physical-world based than magical and mentalistic [7].

The primary limitations of this study and its results include (1) the use of single measures for spirituality, autism spectrum, and schizotypal spectrum traits, (2) the low magnitudes of most of the correlations and regression coefficients, despite high levels of statistical significance, and (3) the use of a highly-educated young Western population. Further joint studies of spirituality in autism and schizotypal traits are warranted, which, given our results, might usefully focus on concepts of God, other aspects of spirituality, the role and nature of searches for meaning through spiritual cognition, and analysis of the psychology of spirituality in non-Western, small-scale, more-traditional populations and cultures. Of particular interest would be studies of cultures where individuals did not differentiate between the 'natural' and 'supernatural', as such groups represent the social-cultural milieu within which spirituality and religion are presumed to have originally evolved.

## References

[1] Barrett JL. Cognitive science of religion: Looking back, looking forward. J Sci Study Relig. 2011; 50(2): 229–239.

[2] Lindeman M, Lipsanen J. Diverse cognitive profiles of religious believers and nonbelievers. Int J Psychol Relig. 2016; 26(3): 185–192.

[3] Lindeman M, Svedholm‐Häkkinen AM. Does poor understanding of physical world predict religious and paranormal beliefs? Appl Cogn Psychol. 2016; 30(5): 736–742.

[4] Norenzayan A, Gervais WM, Trzesniewski KH. Mentalizing deficits constrain belief in a personal God. PLoS One. 2012; 7(5): e36880 10.1371/journal.pone.0036880 22666332 PMC3364254

[5] Jack AI, Friedman JP, Boyatzis RE, Taylor SN. Why do you believe in God? Relationships between religious belief, analytic thinking, mentalizing and moral concern. PLoS One. 2016; 11(3): e0149989 10.1371/journal.pone.0149989 27008093 PMC4805169

[6] Barnes K, Gibson NJ. Supernatural agency: Individual difference predictors and situational correlates. Int J Psychol Relig. 2013; 23(1): 42–62.

[7] Crespi B, Badcock C. 2008; Psychosis and autism as diametrical disorders of the social brain. Behav Brain Scien. 31(3): 241–261. 10.1017/S0140525X08004214 18578904

[8] Jack AI, Dawson AJ, Begany KL, Leckie RL, Barry KP, Ciccia AH, et al fMRI reveals reciprocal inhibition between social and physical cognitive domains. NeuroImage. 2013; 66: 385–401. 10.1016/j.neuroimage.2012.10.061 23110882 PMC3602121

[9] Debbané M, Mohr C. Integration and development in schizotypy research: An introduction to the special supplement. Schizophr Bull. 2015; 41(suppl_2): S363–365. 25810053 10.1093/schbul/sbv003 PMC4373640

[10] Lenzenweger MF. Schizotypy, schizotypic psychopathology and schizophrenia. World Psychiatry. 2018; 17(1): 25–26. 10.1002/wps.20479 29352536 PMC5775125

[11] Fonseca Pedrero E, Debbané M. Schizotypal traits and psychotic-like experiences during adolescence: An update. Psicothema. 2017; 29(1): 5–17. 10.7334/psicothema2016.209 28126052

[12] Fonseca-Pedrero E, Debbané M, Ortuño-Sierra J, Chan RC, Cicero DC, Zhang LC, et al The structure of schizotypal personality traits: A cross-national study. Psychol Med. 2018a; 48(3): 451–462. 28712364 10.1017/S0033291717001829

[13] Fonseca-Pedrero E, Ortuño-Sierra J, Lucas-Molina B, Debbané M, Chan RC, Cicero DC, et al Brief assessment of schizotypal traits: A multinational study. Schizophr Res. 2018b; 197: 182–191. 10.1016/j.schres.2017.10.043 29113776

[14] Breslin MJ, Lewis CA. Schizotypy and religiosity: The magic of prayer. Archive Psychol Relig. 2015; 37(1): 84–97.

[15] Diduca D, Joseph S. Schizotypal traits and dimensions of religiosity. Brit J Clin Psychol. 1997; 36(4): 635–638. 9403156 10.1111/j.2044-8260.1997.tb01270.x

[16] Iyassu R, Jolley S, Bebbington P, Dunn G, Emsley R, Freeman D, et al Psychological characteristics of religious delusions. Soc Psychiatry Psychiatr Epidemiol. 2014; 49(7): 1051–1061. 10.1007/s00127-013-0811-y 24379014 PMC4173112

[17] Jackson M. Benign schizotypy? The case of spiritual experience In Claridge G, editor. Schizotypy: Implications for illness and health. New York, NY: Oxford University Press; 1997 pp. 227–250.

[18] Maltby J, Garner I, Lewis CA, Day L. Religious orientation and schizotypal traits. Pers Indiv Diff. 2000; 28(1): 143–151.

[19] Unterrainer HF, Lewis AJ. The Janus face of schizotypy: Enhanced spiritual connection or existential despair? Psychiatry Res. 2014; 220(1): 233–236. 25086762 10.1016/j.psychres.2014.07.028

[20] Kapur S. Psychosis as a state of aberrant salience: A framework linking biology, phenomenology, and pharmacology in schizophrenia. Am J Psychiatry. 2003; 160(1): 13–23. 10.1176/appi.ajp.160.1.13 12505794

[21] Joseph S, Diduca D. Schizotypy and religiosity in 13–18 year old school pupils. Ment Health Relig Culture. 2001; 4(1): 63–69.

[22] Tobacyk JJ, Wilkinson LV. Magical thinking and paranormal beliefs. J Soc Behav Pers. 1990; 5(4): 255.

[23] Reddish P, Tok P, Kundt R. Religious cognition and behaviour in autism: The role of mentalizing. Int J Psychol Relig. 2016; 26(2): 95–112.

[24] Pennycook G, Cheyne JA, Seli P, Koehler DJ, Fugelsang JA. Analytic cognitive style predicts religious and paranormal belief. Cognition. 2012; 123(3): 335–346. 10.1016/j.cognition.2012.03.003 22481051

[25] Norenzayan A, Gervais WM. The origins of religious disbelief. Trends Cogn Sci. 2013; 17(1): 20–25. 10.1016/j.tics.2012.11.006 23246230

[26] Crespi BJ. The evolutionary etiologies of autism spectrum and psychotic-affective spectrum disorders In Alvergne A, Jenkinson C, Faurie C, editors. Evolutionary thinking in medicine: From research to policy and practice. Oxford, UK: Oxford University Press; 2016 pp. 299–328.

[27] Gleichgerrcht E, Torralva T, Rattazzi A, Marenco V, Roca M, Manes F. Selective impairment of cognitive empathy for moral judgment in adults with high functioning autism. Soc Cogn Affect Neurosci. 2012; 8(7): 780–788. 10.1093/scan/nss067 22689217 PMC3791066

[28] Gray K, Jenkins AC, Heberlein AS, Wegner DM. Distortions of mind perception in psychopathology. Proc Natl Acad Sci USA. 2011; 108(2): 477–479. 10.1073/pnas.1015493108 21187372 PMC3021012

[29] Lindeman M, Svedholm-Häkkinen AM, Lipsanen J. Ontological confusions but not mentalizing abilities predict religious belief, paranormal belief, and belief in supernatural purpose. Cognition. 2015; 134: 63–76. 10.1016/j.cognition.2014.09.008 25460380

[30] Brosnan M, Ashwin C, Walker I, Donaghue J. Can an ‘Extreme Female Brain’ be characterised in terms of psychosis? Pers Indiv Diff. 2010; 49(7): 738–742.

[31] Willard AK, Norenzayan A. “Spiritual but not religious”: Cognition, schizotypy, and conversion in alternative beliefs. Cognition. 2017; 165: 137–146. 10.1016/j.cognition.2017.05.018 28544975

[32] Peoples HC, Duda P, Marlowe FW. Hunter-gatherers and the origins of religion. Hum Nat. 2016; 27(3): 261–282. 10.1007/s12110-016-9260-0 27154194 PMC4958132

[33] Hardt J, Schultz S, Xander C, Becker G, Dragan M. The spirituality questionnaire: Core dimensions of spirituality. Psychol. 2012; 3(01): 116.

[34] Baron-Cohen S, Wheelwright S, Skinner R, Martin J, Clubley E. The autism-spectrum quotient (AQ): Evidence from Asperger syndrome/high-functioning autism, males and females, scientists and mathematicians. J Autism Dev Disord. 2001; 31(1): 5–17. 11439754 10.1023/a:1005653411471

[35] Callaway DA, Cohen AS, Matthews RA, Dinzeo T. Schizotypal Personality Questionnaire—Brief Revised: Psychometric replication and extension. Pers Disord Theory Res Treat. 2014; 5(1): 32. 10.1037/per0000041 24364504

[36] Efron B, Tibshirani RJ. Introduction to the bootstrap New York, NY: Chapman & Hall; 1994.

[37] Fox J, Weisberg S. Companion to applied regression Thousand Oaks, CA: Sage; 2011.

[38] Kim S. ppcor: An R package for a fast calculation to semi-partial correlation coefficients. Comm Stat App Methods. 2015; 22(6): 665–674. 10.5351/CSAM.2015.22.6.665 PMC4681537 26688802

[39] Hammermeister J, Flint M, El-Alayli A, Ridnour H, Peterson M. Gender differences in spiritual well-being: Are females more spiritually-well than males? Am J Health Stud. 2005; 20(2): 80–84.

[40] Bryant AN. Gender differences in spiritual development during the college years. Sex Roles. 2007; 56(11–12): 835–846.

[41] Debbané M, Vrtička P, Lazouret M, Badoud D, Sander D, Eliez S. Self-reflection and positive schizotypy in the adolescent brain. Schizophr Res. 2014; 152(1): 65–72. 10.1016/j.schres.2013.06.027 23819895

[42] Ödéhn N, Goulding A. Schizotypy and mental health in women and men from the general population. Nordic Psychol. 2017; 14: 1–1.

[43] Schjoedt U, Stødkilde-Jørgensen H, Geertz AW, Roepstorff A. Highly religious participants recruit areas of social cognition in personal prayer. Soc Cogn Affect Neurosci. 2009; 4(2): 199–207. 10.1093/scan/nsn050 19246473 PMC2686228

[44] Subbotsky E. Magic and the mind: Mechanisms, functions, and development of magical thinking and behavior Oxford, UK: Oxford University Press; 2010.

[45] Unterrainer HF, Huber HP, Sorgo IM, Collicutt J, Fink A. Dimensions of religious/spiritual well-being and schizotypal personality. Pers Indiv Diff. 2011; 51(3): 360–364.

[46] Raine A. The SPQ: a scale for the assessment of schizotypal personality based on DSM-III-R criteria. Schizophr Bull. 1991; 17(4): 555 1805349 10.1093/schbul/17.4.555

[47] Happé F. Autism: Cognitive deficit or cognitive style? Trends Cogn Sci. 1999; 3: 216–222. 10354574 10.1016/s1364-6613(99)01318-2

[48] Happé F, Frith U. The weak coherence account: Detail-focused cognitive style in autism spectrum disorders. J Autism Dev Disord. 2006; 36(1): 5–25. 10.1007/s10803-005-0039-0 16450045

[49] Kapur S, Mizrahi R, Li M. From dopamine to salience to psychosis—linking biology, pharmacology and phenomenology of psychosis. Schizophr Res. 2005; 79(1): 59–68. 10.1016/j.schres.2005.01.003 16005191

[50] van Os J. ‘Salience syndrome’ replaces ‘schizophrenia’ in DSM‐V and ICD‐11: Psychiatry’s evidence‐based entry into the 21st century? Acta Psychiatr Scand. 2009; 120(5): 363–372. 10.1111/j.1600-0447.2009.01456.x 19807717

[51] Connell A, Koen L, Niehaus D, Cloete KJ, Jordaan E, Botha U. Religious delusions in a Xhosa schizophrenia population. J Relig Health. 2015; 54(5): 1555–1562. 10.1007/s10943-014-9860-0 24711217

[52] Murray ED, Cunningham MG, Price BH. The role of psychotic disorders in religious history considered. J Neuropsychiatry Clin Neurosci. 2012; 24(4): 410–426. 10.1176/appi.neuropsych.11090214 23224447

[53] Fyfe S, Williams C, Mason OJ, Pickup GJ. Apophenia, theory of mind and schizotypy: Perceiving meaning and intentionality in randomness. Cortex. 2008; 44(10): 1316–1325. 10.1016/j.cortex.2007.07.009 18635161

[54] Previc FH. The role of the extrapersonal brain systems in religious activity. Conscious Cogn. 2006; 15(3): 500–539. 10.1016/j.concog.2005.09.009 16439158

[55] Caldwell-Harris C, Murphy CF, Velazquez T, McNamara P. Religious belief systems of persons with high functioning autism. Cogn Sci. 2011; 33(33): 3362–3366.

[56] Bering JM. The existential theory of mind. Rev Gen Psychol. 2002; 6(1): 3.

